# Electron Beam Immobilization of Novel Antimicrobial, Short Peptide Motifs Leads to Membrane Surfaces with Promising Antibacterial Properties

**DOI:** 10.3390/jfb9010021

**Published:** 2018-02-27

**Authors:** André Reinhardt, Isabell Thomas, Julie Schmauck, Ralf Giernoth, Agnes Schulze, Ines Neundorf

**Affiliations:** 1 Department of Chemistry, Biochemistry, University of Cologne, Zülpicher Str. 47a, D-50674 Cologne, Germany; andre.reinhardt1@gmx.de; 2 Leibniz Institute of Surface Engineering, Permoserstr. 15, D-04318 Leipzig, Germany; isabell.thomas@iom-leipzig.de; 3 Department of Chemistry, Organic Chemistry, University of Cologne, Greinstr. 4, D-50939 Cologne, Germany; jpieperg@smail.uni-koeln.de (J.S.); ralf.giernoth@uni-koeln.de (R.G.)

**Keywords:** biofilm formation, electron beam, antimicrobial peptides, surface modification, immobilization techniques

## Abstract

In this study, the efficacy of electron beam irradiation versus chemical coupling for yielding polyethersulfone (PES) membranes with antibacterial properties was investigated. For the surface coating, a recently discovered lead compound, IL-KKA, comprising a short peptide sequence functionalized with imidazolium groups, was used. For better integration within the membrane, several novel variants of IL-KKA were generated. Membrane immobilization was achieved using different doses of electron beam irradiation and NHS/EDC chemical coupling. Physicochemical characterization of the coated membranes was performed by water contact angle measurements, X-ray photoelectron spectroscopy, and scanning electron microscopy. Our results show that electron beam irradiation is as effective and gentle as chemical coupling using the NHS/EDC method. Moreover, it was demonstrated that the obtained membranes exhibit promising antibacterial activity against *B. subtilis*. In summary, the technique presented herein might be promising as a template for developing future anti-biofilm devices.

## 1. Introduction

Biofilm-associated infections on medical devices represent a serious public health problem. Biofilms are well-organized microbial communities found on abiotic surfaces or tissue. Herein, the organisms are encased in a self-produced slimy polymer matrix [1], which is composed of extracellular polysaccharides, nucleic acids, glycoproteins and water. The formation of these sessile communities occurs during four developmental stages, beginning with the adherence of microorganism to the surface. This is followed by production of the extracellular layer leading to a dense aggregation of the organisms. Then biofilm maturation takes place, including formation of water channels and other complex structures. Biofilm spreading results as a final step, in which adjacent regions become colonized [2,3,4]. With the generation of such a complex biofilm matrix, the organisms protect themselves against environmental stresses such as UV radiation, pH variation, and osmotic shock. Notably, biofilm-growing bacteria count as the main reason for many chronic bacterial infections, and are responsible for persistent infections, which are often accompanied by the development of resistances [5]. Furthermore, within biofilms, the resistance against antibiotics is up to 10–1000 times higher, when compared to the planktonic state of the same bacteria [6,7]. It is estimated that approximately 80% of human bacterial infections are caused by biofilm formation on medical devices such as implants, catheters, and heart valves [8]. Beside biofilm formation on medical devices, biofouling is also an important issue for nanocomposite membranes that are used for wastewater treatment and desalination. In this respect, membranes with antimicrobial properties have garnered widespread attention as a promising strategy to mitigate biofouling processes and are highly appreciated [9]. To prevent biofilm formation by microbial pathogens, several strategies have been reported [10,11,12,13]. Substantial research has been performed using different coatings, e.g., colonizing surfaces with non-pathogenic bacteria [6], and/or coating surfaces with biocidal substances like triclosal or polyquaternary amines [14,15,16]. A matter of much interest is, furthermore, the direct immobilization of antimicrobial compounds, such as vancomycin, penicillin, or ampicillin on medical devices [17,18,19,20].

In recent years, antimicrobial peptides (AMPs) have also gained much attention as an alternative to conventionally used drugs [21]. AMPs are host defense molecules present in nearly all organisms, ranging from bacteria to humans [22]. Interestingly, their main mechanism of action includes permeabilization of the membrane, making it difficult for the pathogen to develop resistances [23]. Many AMPs are easily synthesized chemically in a cost-effective way. This allows insertion of various modifications that usually do not harm, but often improve their activity spectra [24]. Moreover, AMPs are increasingly being investigated as antibiotic surface coatings [25].

Within this work, we describe the surface modification of polyethersulfone (PES) membranes with short antimicrobial peptide-conjugates using two different methods. PES membranes have gained increasing importance in diverse filtration applications, e.g., in hemodialysis, water filtration, or sterilization filtration [26]. Furthermore, enzyme-membrane bioreactors are used for the synthesis of pharmaceuticals or fine chemicals. Here, the membrane is used as support material for the immobilized enzyme and simultaneously separates the starting material from product [27,28,29,30,31]. The diverse applications often require a surface functionalization of the polymer membrane to avoid fouling or biofouling, to improve the hydrophilicity, or to introduce special functional groups [32,33,34]. In previous studies, electron beam (E-Beam) irradiation was presented as a versatile tool for easy immobilization of small organic molecules [35,36], polymers [37], or even enzymes [38,39,40,41] on polymer membranes. This method is now used for the first time to immobilize short antimicrobial peptide-conjugates on a PES membrane.

**Figure 1:**
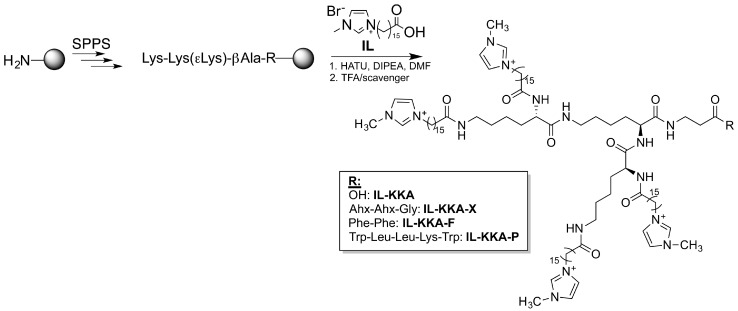
Synthesis scheme and structure of the novel IL-KKA-based peptides that were used within this study. (SPPS: solid-phase peptide synthesis; HATU: [*O*-(7-azabenzotriazol-1-yl)-*N*,*N*,*N*′,*N*′-tetramethyluronium-hexafluorophosphate]; DIPEA: diisopropylethylamine; DMF: dimethylformamide; TFA: trifluoroacetic acid).

The conjugates presented are composed of a short amino acid sequence modified with four functionalized imidazolium groups [42,43]. Recently, we demonstrated that the lead compound, namely **IL-KKA** (cf. Figure 1), exhibited highly promising antimicrobial activity even against drug-resistant strains [44]. The functionalized membranes were characterized concerning the differently used immobilization strategies, and their physical as well as biological properties.

## 2. Results and Discussion

### 2.1. Synthesis of New IL-KKA Variants

Three new variants of the already described **IL-KKA** peptide were synthesized that all differed in their *C*-terminal linker structure. Thus, peptides with a more flexible linker Ahx-Ahx-Gly, containing two aminohexane moieties (Ahx: 6-(amino)hexanoic acid) (**IL-KKA-X**), or peptides with a more hydrophobic linker containing two phenylalanine residues (**IL-KKA-F**), as well as peptides coupled to a short antimicrobial amino acid sequence comprised of the residues Trp-Leu-Leu-Lys-Trp (**IL-KKA-P**) [45] (Figure 1, Table 1) were generated. The novel *C*-terminal modifications should promote a better integration within the PES membrane during the irradiation process, e.g., by increasing the distance to the surface, as in the case of **IL-KKA-X**, or by supporting hydrophobic interactions with the surface as in the case of **IL-KKA-F**. Moreover, by introduction of an additional hydrophobic antimicrobial peptide (AMP) sequence, the overall antimicrobial activity is thought to increase by synergistic effects.

All peptide conjugates were synthesized according to a previously reported method [44]. Wang resin was used, beginning with the short *C*-terminal peptide sequence. Finally, the imidazolium groups were coupled using standard solid-phase peptide synthesis (SPPS) coupling methods. Cleavage from the solid support was performed by adding a solution of concentrated trifluoroacetic acid and scavenger. All peptides were purified and analyzed using reversed phase HPLC coupled to an ESI-mass spectrometry device. Analytical data is presented in Table 1.

### 2.2. Electron Beam Modification of PES Membranes

**Figure 2:**
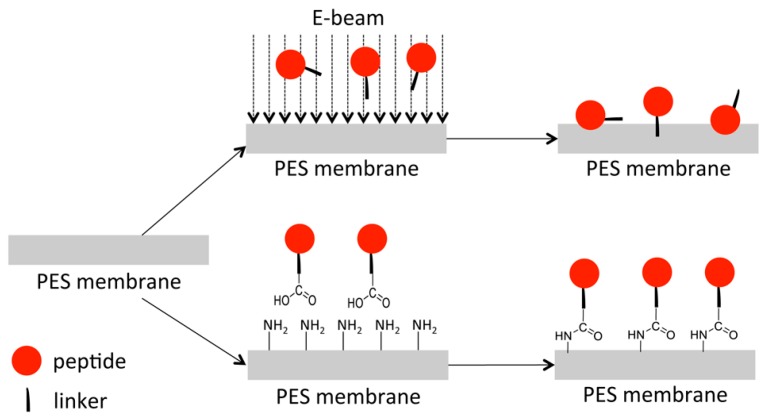
Schematic illustration of the two different immobilization strategies applied: using either electron beam irradiation or chemical coupling, respectively. For NHS/EDC activation, the PES membrane was activated with 2-aminoethylmethacrylate hydrochloride to obtain an amino functionalized surface.

The four different IL-KKA compounds were covalently immobilized to PES membranes using either electron beam (EB) irradiation of different doses (50, 100, 150 kGy, respectively), or chemical coupling. In the latter case, the PES membranes had to be modified with amino groups first. This was achieved by functionalization of the membrane surface with 2-aminoethylmethacrylate hydrochloride via EB irradiation (150 kGy). Subsequently, the amino functionalized PES membranes were converted with the *C*-terminal of the peptides by NHS/EDC activation (Figure 2). The E-Beam irradiation leads to the formation of radicals, which can undergo recombination reactions to form non-specific covalent bonds with the peptide. The second approach results in a specific covalent immobilization of the peptide via a linker on the membrane surface. Both techniques lead to the formation of covalent bonds.

Successful immobilization on the membrane surface was confirmed by physicochemical and structural characterization using X-ray photoelectron spectroscopy (XPS), contact angle measurements, as well as scanning electron microscopy (SEM). XPS of the membrane surface area (Table 2) revealed that the unmodified PES membrane mainly consists of carbon (69.95%), oxygen (26.12%), and sulfur (3.93%). After the immobilization step, a significant increase in nitrogen on the outermost membrane surface was detected, regardless of the compound that was attached. Since the membrane polymer itself does not contain any nitrogen, this effect can be assigned to the successful modification and presence of the peptides on the membrane surface. Comparing the nitrogen content for each compound and immobilization technique, two trends can be highlighted. On the one hand, immobilization by an EB dose of 150 kGy seemed to be more successful for the two compounds **IL-KKA-F** (1.34%) and **IL-KKA-P** (1.73%) compared to chemical coupling with NHS/EDC (**IL-KKA-F**: 0.33%, **IL-KKA-P**: 0.73%). This observation might be the result of the more hydrophobic *C*-terminal part of both compounds leading to a better interaction with the hydrophobic surface of the PES membrane during the irradiation process. For the other two conjugates, **IL-KKA** and **IL-KKA-X**, the highest nitrogen values were obtained after chemical coupling (**IL-KKA**: 0.98%; **IL-KKA-X**: 1.03%). So it can be assumed that these two peptides do not interact that effective with the membrane during the EB immobilization.

Interestingly, the data from the contact angle measurements perfectly fit with these observations. After EB-mediated functionalization of the PES membrane with **IL-KKA-F** and **IL-KKA-P**, respectively, the highest contact angles were obtained, pointing to an increase in hydrophobicity on the membrane surface. Since **IL-KKA-P** contains more hydrophobic amino acids, the values increased significantly above 60° (**IL-KKA-P**: 68.1° vs. **IL-KKA-F**: 61.5°). Notably, for all **IL-KKA** variants, an increase in contact angles is observed depending on the E-beam dose applied. Generally, the values obtained with the NHS/EDC method were in every case relatively low compared to the E-beam values. This might point to a smaller amount of compound being integrated on the membrane, and the presence of free, uncoupled amino groups, making the membrane surface more hydrophilic. However, the resulting peptide activity might be different because immobilization was performed via a non-specific immobilization method, and therefore, the active part of the peptide might be not accessible at the membrane surface.

**Figure 3:**
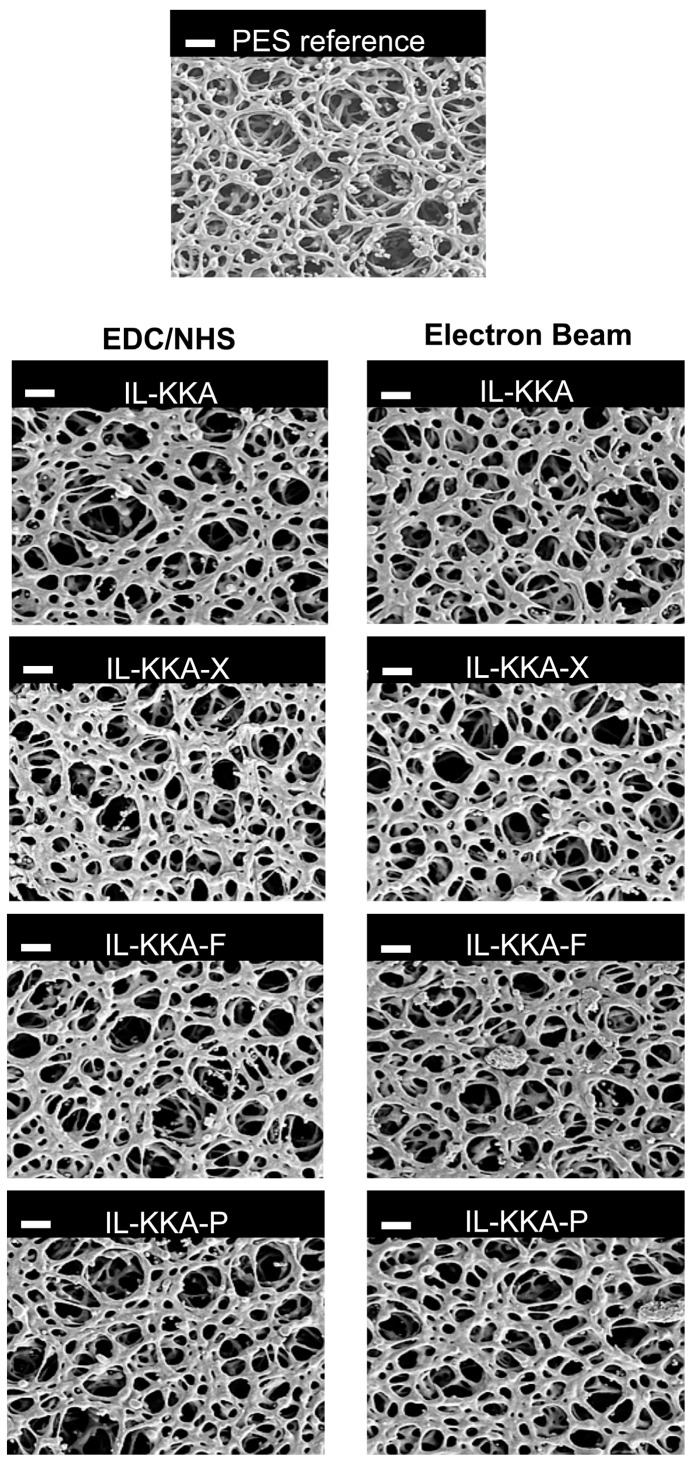
SEM images of PES membranes: reference membrane; after coupling with EDC/NHS (**left**), and after electron beam immobilization (**right**) of **IL-KKA**, **IL-KKA-X**, **IL-KKA-F**, and **IL-KKA-P**, respectively.

To analyze the surface cross-sectional area morphology of the PES membranes, we performed SEM imaging (see Figure 3). The reference PES membrane is highly porous with an open sponge-like pore structure. After electron beam immobilization of the different peptides this structure is not negatively affected. The pore structure remains open, and no pore blocking by the peptides or structural defects caused by the irradiation treatment were detected (Figure 3). These results are in good accordance with previous studies regarding the electron beam immobilization of enzymes on polymer membranes [37,38,39,40]. Here, the large biomolecules could be attached to the membrane surface and no significant changes regarding the membrane performance, e.g., pore size, porosity, and water permeation flux, were detected. This can be explained by the fact that the protein cover layer, which is immobilized on the membrane surface, is very thin compared to the pore size.

### 2.3. Antimicrobial Activity

**Figure 4:**
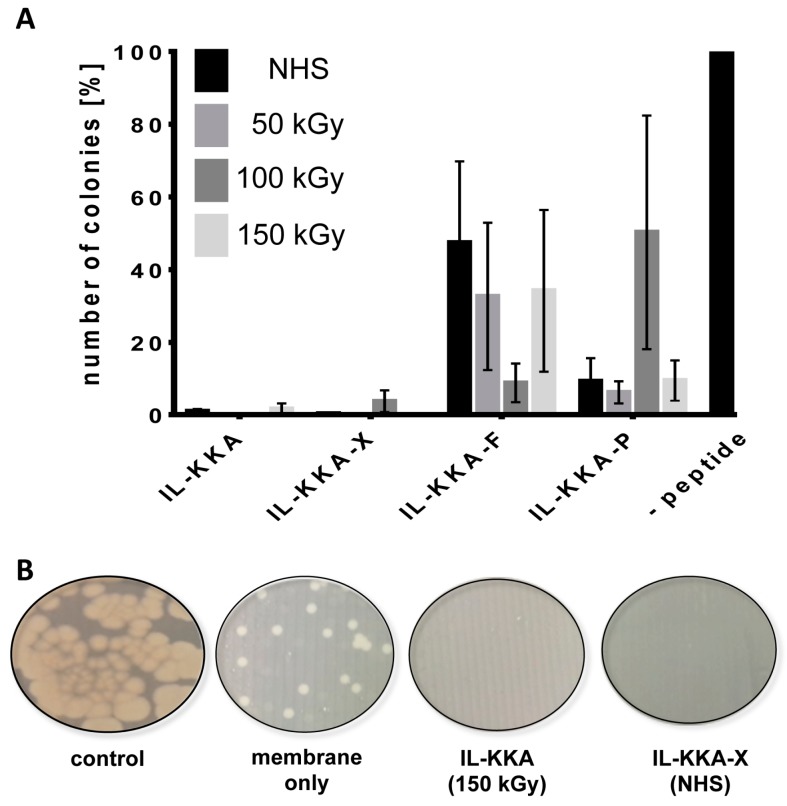
(**A**) Antimicrobial activity assay against *B. subtilis* (*n* = 3)*.* PES membranes without peptides were used as a negative control and were set to 100%. Four different peptides, **IL-KKA**, **IL-KKA-X**, **IL-KKA-F** and **IL-KKA-P** were tested with different immobilization methods. NHS = covalent coupling via a peptide bond. (kGy = kGray) (**B**) Examples of distinct extracts of the agar plates treated with bacterial solutions that were incubated w/o membrane or any of the peptides (control), membrane only, or **IL-KKA** and **IL-KKA-X**, respectively.

Next, we tested whether the membrane surface would have any influence on bacterial colonization. Therefore, the respective membranes were incubated with a solution containing *Bacillus subtilis* as a representative bacterial strain. After 3 h incubation, a small amount of this solution was exposed on agar plates. The organisms were grown for an additional 24 h, and afterwards, all developed colonies were counted. Figure 4A shows that all membranes that were coated with the different variants exhibited an increased activity against *B. subtilis* compared to membranes tested without peptide. Moreover, it was observed that membranes coated with **IL-KKA** or **IL-KKA-X** showed a more pronounced and significant inhibition of bacterial growth. In this case, almost no colonies grew on the agar plates as can be seen from Figure 4B. Considering the supposed higher amount of **IL-KKA-P** and **IL-KKA-F** on the outermost surface of the membrane, this observation is somehow unexpected. Additionally, for all investigated variants, it seems that their activity is independent of the immobilization technique used. Both facts might be explainable by the non-specific immobilization technique utilized, whereby the peptide-conjugates become randomly attached to and within the PES membrane. Obviously, **IL-KKA** or **IL-KKA-X** are not affected in their activity at all when immobilizing them on surfaces.

## 3. Materials and Methods

### 3.1. Chemicals and Materials

Polyethersulfone (PES) membranes (Durapore, pore size 0.2 μm, Millipore, Darmstadt, Germany) were purchased from Carl Roth GmbH & Co. (Karlsruhe, Germany). 2-Aminoethyl methacrylate hydrochloride (AEMA), *N*-hydroxysuccinimide (NHS), TIS (triisopropylsilane), DIC (*N*,*N*′-diisoproylcarbodiimide), HATU (1-[bis(dimethylamino)methylene]-1H-1,2,3-triazolo[4,5-b]pyridinium 3-oxid hexafluorophosphate), DMF (dimethylformamide), piperidine, DIPEA (*N*,*N*-diisopropylethylamine), and 1-ethyl-3-(3-dimethylaminopropyl)carbodiimide (EDC) were purchased from Sigma Aldrich (St. Louis, MO, USA). Oxyma pure^®^ and all Fmoc (fluorenylmethoxycarbonyl)-protected amino acids were purchased from Iris Biotech (Marktredwitz, Germany). The used water was ultrapure water taken from a MilliQ-System (Billerica, MA, USA). All chemicals were analytical grade and used without further purification.

### 3.2. Solid-Phase Peptide Synthesis

Peptides were synthesized according to ref [44]. Briefly, Wang resin beads (substitution 1.1 mmol/g, 0.015 mmol scales) were coupled manually with the first amino acid overnight using 5 eq. Fmoc-aa-OH, Oxyma pure^®^ and DIC (*N*,*N*′-diisoproylcarbodiimide). The following amino acids were coupled with an automated peptide synthesizer from MultiSynTech using double coupling steps with 8 equivalents (eq.). Fmoc-aa-OH, Oxyma pure^®^ and DIC. Fmoc-protecting group was removed with piperidine (20% in DMF (dimethylformamide), 5 min followed by 20% in DMF, 15 min). The imidazolium-salts [(CO_2_H)^15^C_15_C_1_im]Br (Figure 1) were coupled manually on resin using HATU (1-[bis(dimethylamino)methylene]-1H-1,2,3-triazolo[4,5-b]pyridinium 3-oxid hexafluorophosphate) and DIPEA (*N*,*N*-diisopropylethylamine) as activating reagents in the same protocol as stated above [44]. In all cases, synthesis progress was monitored by ninhydrin colorimetric test and by reversed phase (RP)-HPLC-electron spray ionization (ESI) mass spectrometry (MS) after sample cleavage.

After successful synthesis, peptides were removed from the resin using TFA (trifluoroacetic acid)/TIS (triisopropylsilane)/H_2_O (95:2.5:2.5 *v*/*v*/*v*) for 3 h, and precipitated in ice-cold diethyl ether. Peptides were purified using preparative RP-HPLC, and fractions were analyzed by analytical RP-HPLC ESI-MS. Peptide-containing fractions were combined and lyophilized. Final purity of all compounds was >95% (see, Table 1).

### 3.3. Immobilization of Different Peptides on PES Membranes

For peptide immobilization, the PES membrane disc (Ø 10 mm) was immersed in a peptide solution (2.5 mg/mL in ultrapure water) for 5 min, followed by electron beam irradiation (E-Beam) with a dose of 50, 100, or 150 kGy, respectively, according to a previously published method [38,40]. Irradiation was performed in a N_2_ atmosphere with O_2_ quantities <10 ppm. The voltage and the current were set to 160 kV and 10 mA, respectively. Then, the irradiated membrane was rinsed 3 × 30 min with ultrapure water and dried at ambient temperature. The E-Beam treatment results in the formation of ions, excited molecules and free radicals, as described for the radiolysis of water. Thus, the activation of both the dissolved peptide as well as of the membranes will be accomplished. The formed radicals/activated species can undergo various reactions, such as cross-linking or recombination reactions. This way, links between the polymer matrix and the peptide can be formed.

Alternatively, peptides were immobilized by a chemical linker system. For this purpose, the membranes had first to be functionalized with amino groups at the surface. The PES membrane disc (Ø 47 mm) was immersed in a solution of AEMA (2-aminoethyl methacrylate hydrochloride) (0.5 wt. % in water) for 30 min, followed by E-Beam irradiation (150 kGy). Then, the samples were washed with water (3 × 30 min) and dried at ambient temperature. The membranes were cut into 10 mm discs and were treated with an aqueous solution of the peptide (2.5 mg/mL), NHS (*N*-hydroxysuccinimide) (5 mg/mL), and EDC (1-ethyl-3-(3-dimethylaminopropyl)carbodiimide) (5 mg/mL). The coupling was allowed to react overnight at room temperature. Then, membranes were washed 3 × 30 min with ultrapure water and dried at ambient temperature.

### 3.4. Membrane Characterization

The membrane morphology was investigated by scanning electron microscopy (SEM, Ultra 55, Carl Zeiss SMT, Jena, Germany). In order to prevent charging, the sample was sputtered with a thin gold layer.

The chemical surface composition was analyzed with X-ray photoelectron spectroscopy (AXIS Ultra, Kratos Analytical, Manchester, UK). The kinetic energy of the electrons was analyzed with a pass energy of 160 eV for the survey spectra and 40 eV for the energy resolved spectra, respectively. Elements were identified from the survey spectra.

The water contact angle of the membrane samples was determined with the sessile drop method on a DSA 30E (Krüss, Germany). A drop (5 µL) of water was placed on the membrane with a microsyringe. At least ten contact angles per five different locations were averaged.

### 3.5. Antimicrobial Activity

For antimicrobial activity tests, the Gram-positive bacterium (Bacillus subtilis (ATTC 6633)), was used as test strain. The bacteria were cultured in Mueller-Hinton Broth (MHB) overnight at 37 °C and diluted to an OD_600_ = 0.001 (corresponding to 10^6^ bacteria/mL). PES membranes with peptides (control: membrane without peptide) were put at the bottom of a 48 well-plate. In each well, 50 μL of bacteria suspension was added, and the solution was incubated for 3 h at 37 °C. Bacteria suspensions were diluted 1:100 with PBS and 10 µL were exposed onto Mueller-Hinton agar plates. The next day, the grown bacteria colonies were counted, whereas the control membrane without peptide was set to 100%. All experiments were done in triplicate with *n* = 3.

## 4. Conclusions

In conclusion, we were able to show that electron beam irradiation is an effective immobilization method, and reaches the applicability of chemical coupling methods. Although the E-Beam process should be adjusted to each molecule that is investigated, our results clearly demonstrate successful functionalization of membranes with bioactive compounds. Moreover, since E-Beam modification proceeds in only one step, it overcomes the need of previous functionalization steps. Additionally, no initiators, catalysts, or organic solvents are required. Using this method, membrane surfaces with antibacterial activity against *B. subtilis* were synthesized by immobilization of short peptides that have been demonstrated to show activity even against drug-resistant strains. While the membrane coating resulted in antibacterial properties, membrane performance properties regarding pore size, porosity, and permeation flux were not influenced. These findings indicate that a very thin layer of peptide was coated on the membrane surface.

All in all, this easy and powerful immobilization method might be highly useful for the future to covalently immobilize antimicrobial compounds on polymeric devices.

## Figures and Tables

**Table 1:** Names, sequences and analytical data of all peptide conjugates. (The gradient was 10–60% acetonitrile in water in 15 min with 0.1% formic acid, except for IL-KKA, where the gradient was 10–60% in 15 min with 0.1% TFA.). βA: beta-alanine; Ahx: 6-aminohexanoic acid.

Name	Sequence	MW_calc_ [Da]	MW_exp_ [Da]	Net Charge	RT [min]	Purity [%]
**IL-KKA**	IL_4_-KK(εK)βA-OH	1751.7	1752.2	+4	18.78	98
**IL-KKA-X**	IL_4_-KK(εK)βA-Ahx-Ahx-Gly-OH	2035.1	2035.7	+4	15.63	98
**IL-KKA-F**	IL_4_-KK(εK)βA-Phe-Phe-OH	2046.1	2046.8	+4	15.08	95
**IL-KKA-P**	IL_4_-KK(εK)βA-Trp-Leu-Leu-Lys-Trp-OH	2478.7	2479.6	+5	13.75	97

**Table 2:** Atomic composition of the membranes as determined by X-ray photoelectron spectroscopy (XPS) and contact angle (CA) measurements.

	Elemental Ratio (Relative Atom-%)				CA [°]
Label	C	N	O	S	
**PES Membrane (reference)**	69.95	-	26.12	3.93	55.3
**Membrane + IL-KKA 50 kGy**	67.81	0.69	28.17	3.33	45.2
**Membrane + IL-KKA 100 kGy**	69.25	0.25	26.87	3.62	53.5
**Membrane + IL-KKA 150 kGy**	69.39	0.4	27.05	3.56	60.4
**Membrane + IL-KKA (NHS/EDC)**	69.61	1.02	26.61	3.78	44.8
**Membrane + IL-KKA-X 50 kGy**	69.28	0.71	26.85	3.17	54.2
**Membrane + IL-KKA-X 100 kGy**	69.44	0.69	26.61	3.27	56.4
**Membrane + IL-KKA-X 150 kGy**	68.79	0.17	27.47	3.56	58.7
**Membrane + IL-KKA-X (NHS/EDC)**	69.42	1.03	26.50	3.06	50.7
**Membrane + IL-KKA-F 50 kGy**	68.38	0.81	27.53	3.28	48.1
**Membrane + IL-KKA-F 100 kGy**	67.84	0.59	28.20	3.37	60.2
**Membrane + IL-KKA-F 150 kGy**	69.55	1.34	25.86	3.25	61.5
**Membrane + IL-KKA-F (NHS/EDC)**	68.95	0.33	27.06	3.65	46.1
**Membrane + IL-KKA-P 50 kGy**	68.81	0.72	27.12	3.35	58.0
**Membrane + IL-KKA-P 100 kGy**	68.60	0.73	27.34	3.33	58.1
**Membrane + IL-KKA-P 150 kGy**	70.51	1.73	24.93	2.85	68.1
**Membrane + IL-KKA-P (NHS/EDC)**	69.15	0.73	26.79	3.33	49.5
